# Obstetric Danger Signs: Knowledge, Attitude, Health-Seeking Action, and Associated Factors among Postnatal Mothers in Nekemte Town, Oromia Region, Western Ethiopia—A Community-Based Cross-Sectional Study

**DOI:** 10.1155/2020/6573153

**Published:** 2020-09-01

**Authors:** Misganu Teshoma Regasa, Jote Markos, Ashenafi Habte, Shivaleela P. Upashe

**Affiliations:** ^1^Department of Midwifery, Institute of Health Sciences, Wollega University, Nekemte, Ethiopia; ^2^Department of Nursing, Institute of Health Sciences, Wollega University, Nekemte, Ethiopia; ^3^Department of Pediatrics and Neonatal Nursing, Institute of Health Sciences, Wollega University, Nekemte, Ethiopia

## Abstract

**Background:**

Maternal mortality remains unacceptably high due to pregnancy complications and remains the major health problems in many developing countries such as Ethiopia. Having poor knowledge of obstetric danger signs contributes to delays in seeking and receiving skilled care which in turn increases maternal mortality. However, in Ethiopia, studies are lacking regarding the knowledge level of mothers about obstetric danger signs during pregnancy, child birth, and postnatal periods. In Ethiopia, the proportion of those who have full knowledge of these obstetric danger signs during pregnancy, child birth, and postnatal period is not known. Despite few studies are conducted at health facility level focusing on danger signs during pregnancy, the issue of health-seeking action after identifying danger signs and attitude of mothers towards obstetric danger sign was not addressed.

**Objectives:**

To determine knowledge, attitude, health-seeking action towards obstetric danger signs, and associated factors among postpartum women.

**Methods:**

A community-based cross-sectional study was conducted in Nekemte Town from October 1 to November 30, 2017. Multistage sampling technique was employed to select the total sample size of 621. Ethical clearance was obtained from Wollega University research and ethical committee. A pretested structured questionnaire was used to collect data from respondents. Data were entered to EpiData version 3.1 and exported to SPSS version 20 for analysis. To assess the associations between dependent and independent variables, binary and multivariate logistic regressions were employed, and the strength of association was presented using odds ratios with 95% confidence intervals.

**Result:**

Only 197 (32.3%) of respondents were able to spontaneously mention at least five key obstetric danger signs during antepartum, intrapartum, and postpartum (in the three phases) with at least one obstetric danger sign in each phase and thus were considered as having good knowledge of key obstetric danger signs. Government employee (AOR = 3.28, 95% CI: 1.98–5.42), able to read and write (AOR = 4.92, 95% CI: 2.14–11.3), primary school (AOR = 4.90, 95% CI: 2.11–11.4), ANC follow-up (AOR = 6.2, 95% CI: 1.82–21.21), and ANC visit (AOR = 4.07, 95% CI: 2.35–7.06) were significantly associated with knowledge of obstetric danger sign. From 150 (24.6%) participants who faced obstetric danger signs during their last pregnancy, the majority of them, 137 (91.3%), had a good practice which is seeking a health facility for care. *Conclusion and Recommendation*. Despite their low knowledge level and attitude, the practice of mothers in response to obstetric danger signs was encouraging. Occupation, educational status, ANC follow-up, and number of ANC visits were variables significantly associated with knowledge of obstetric danger signs. Health care providers should provide health education and counseling to increase awareness, and appropriate counseling during antenatal care at each visit is of paramount importance.

## 1. Background

Most cases of pregnancy-related complications are unpredictable that could result in the death of the mother and infant [1]. Globally, the majority (80%) of maternal deaths are happening due to direct obstetric complications. These includes hemorrhage, unsafe abortion, pregnancy induced hypertension, infection, and obstructed and prolonged labor [2].With the assumption that “every pregnancy faces risks, “women should be made aware of obstetric danger sings during pregnancy, delivery, and postpartum period [1]. Obstetric danger sign can be classified into danger signs during pregnancy, child birth, and postpartum. Common danger signs during pregnancy include severe vaginal bleeding, blurred vision, and swollen hands/face, and common danger signs during child birth include severe vaginal bleeding, retained products of concept tissue/retained placenta, convulsions, prolonged labor (>12 hours), and major danger signs during postpartum includes foul-smelling vaginal discharge, severe vaginal bleeding, and fever [3]. Lack of awareness about obstetric danger signs contributes to delays in seeking and receiving skilled care [3]. Subsequently, early recognition of these danger signs is very important in order to avoid delay in decision to seek health care, the second most important contributor of maternal mortality [4].

Even though health-seeking action was poor among adolescents as compared to adults, the majority of the women (75.3%) were having appropriate health-seeking action after recognizing a danger sign in their last pregnancy [5, 6]. Most of the studies have shown poor knowledge of obstetric danger signs not only during pregnancy but also during delivery and after childbirth [7]. Young women/adolescent mothers were more likely to attend antenatal care visits less than four times as compared to adult women and hence miss an opportunity to be educated on danger signs during pregnancy, delivery, and postdelivery [8]. Likewise, in Bangladesh, young mothers were less likely to seek healthcare at the time of child birth [9].

The study conducted in Turkey indicated that cultural influences were linked to delay in seeking care when they experience complications and this was contributed by factors such as age, educational level, health insurance, getting antenatal care, family structure, and knowledge of the danger signs during pregnancy. This shows the impact of knowledge on health-seeking actions among pregnant women [10].

A cross-sectional study conducted in Arba Minch town revealed that age, educational status, family income, and decision-making power were significantly associated with obstetric danger sign knowledge [11]. Visiting a health facility for care is the best way to identify any problem that may endanger the health of the mother/unborn baby and take actions early and promptly [12].

Poor knowledge is compounded by the limited access in rural areas and less-skilled health workers that are available in rural areas, but the problem still persists even in urban areas where it is acknowledged to have more health facilities, improved infrastructure, and transport. Irrespective of the cost and easier access to health facilities, a low level of knowledge is also highly likely caused by inadequate dissemination of information on danger signs at the health facilities [2].

Having knowledge of obstetric danger sign is a crucial step to get an appropriate and timely obstetric care. However, in Ethiopia, the proportion of those who have full knowledge of these obstetric danger signs is not known. Likewise, knowledge alone is not a guarantee to seek care but also attitude of the mother's matter. However, attitude and health-seeking actions after identifying obstetric danger signs were not investigated. Previous studies mostly done at health facility level focused only on danger signs during pregnancy. However, these studies tried to assess obstetric danger signs at each three phases, during pregnancy, child birth, and postpartum at community level, and provided insight information on pregnant women's knowledge, attitude, and health-seeking actions about obstetric danger signs in the study area, which could help in designing appropriate interventions.

## 2. Objectives of the Study

### 2.1. General Objective

- To determine knowledge, attitude, health-seeking actions towards obstetric danger signs, and associated factors among postpartum women

### 2.2. Specific Objectives

- To determine the knowledge of Obstetric danger signs among postpartum women
- To assess the mother's attitude towards obstetric danger signs among postpartum women
- To assess factors affecting knowledge of obstetric danger signs among postpartum women
- To assess health-seeking actions among postpartum women after they faced obstetric danger signs

## 3. Methodology

### 3.1. Study Area and Period

The study was conducted in Nekemte Town, East Wollega Zone, Oromia Region. Nekemte is the capital town of East Wollega Zone located at 331 km away from Addis Ababa, the capital city of Ethiopia. According to Nekemte Town administration report, the total population in 2016/2017 is 117,771. The town is divided into eight administrative kebeles. The study was conducted from October 1 to November 30, 2017.

### 3.2. Study Design

A community-based cross-sectional study design was conducted.

### 3.3. Population

#### 3.3.1. Source Population

All women who gave birth at least once in the last one year preceding the study irrespective of place and outcome of delivery.

#### 3.3.2. Study Population

Randomly selected women who gave birth in the past one year prior to the study.

## 4. Eligibility Criteria

### 4.1. Inclusion Criteria

All mothers who gave birth at least once in the past one year prior to the study regardless of their birth outcome and residing in the study area for at least six months.

### 4.2. Exclusion Criteria

Mothers who did not give response due to different illness such as critically ill mothers and women with mental health problem were excluded from the study.

## 5. Sample Size and Sampling Techniques

### 5.1. Sample Size Determination

The sample size was determined using single population proportion formula considering the following assumptions:

  Previously, a study done in Arba Minch Town indicates *p*=24.1% (proportion of knowledge of obstetric danger signs [11]
  Confidence interval, 95%, *Zα*/2 = 1.96
  Margin of error, 5%, *D* = 0.05.
  Design effect (*d*) = 2
 
*N* = 3.8416
   **N** **=** **282**
  The final sample size was adjusted by assuming design effect of 2 and 10% nonresponse rate
  Nf = 564 + 56 = 621

### 5.2. Sampling Techniques and Procedure

A multistage sampling technique was employed. From the seven kebeles, four kebeles were selected by simple random sampling methods using lottery methods. Household enumeration was carried out; households were chosen after a random start at a central place in the village. The direction was selected at random by spinning a pen, and the data collectors walk to the edge of the village in the direction that the pen pointed, numbering all households. Each household was given an identification number which was later used as the sampling frame. Systematic sampling technique of every 4^th^ interval was used to take households from each of the selected 4 kebeles by taking into account the number of households in each of the sampled kebeles until the calculated sample size in the respective kebeles was reached to achieve the sample size of 621 households in total. The *k* interval, i.e., the 4^th^ interval was determined by dividing the total eligible households within the sampled kebeles (3037 households) by the total sample size (621). Proportional sample size allocation was performed for each selected kebele. Proportional sample size allocation was done by multiplying the number of eligible households in each kebele by sample size and dividing it by the total number of eligible households. Accordingly, the total number of households having eligible women was 3037 (902, 705, 610, and 820 in Darge, Kase, Burka Jato, and Dune Kane kebeles, respectively).Hence, 185, 144, 125, and 167 households were taken from Darge, Kase, Burka Jato, and Dune Kane kebeles, respectively. The index household was then selected by simple random sampling using the lottery method, and subsequently, the sampling interval was used to select the next household. Immediately, the next household was selected whenever eligible women were not found in the 4^th^ household. Every time more than one eligible women were encountered in one household, and one woman was selected by the lottery method.

### 5.3. Data Collection Procedure

Data were collected by a face-to-face interview using a structured and pretested questionnaire (Supplementary (available here)) which was first prepared in English and translated to Afan Oromo which is the official language of the region prior to the start of the fieldwork. Twelve Urban health extension workers who are fluent in the official language of the region collected the data from October 1 to November 30, 2017. One supervisor with experience in community health service provision supervised the data collection process. Both the data collectors and supervisor were given two days of comprehensive training before the actual work about the aim of the study, procedures, and data collection techniques going through the questionnaires question by question, art of interviewing, ways of collecting the data, and clarification was given by the investigators. The issue of confidentiality and privacy was focused during the training session, and they have participated in pretesting of the questionnaire after their training.

### 5.4. Data Quality Control

The quality of data was assured by proper designing and pretesting of the questionnaires in Gimbi Town on 5% (32) of participants, to ensure that the questions are clear and can be understood by both data collectors and the respondents, and further questionnaires were refined based on the results. Training was given for the data collectors and supervisors before the actual data collection. Every day after data were collected, questionnaires were reviewed and checked for completeness and relevance by the supervisors and principal investigators, and the necessary feedback was offered to data collectors in the next morning.

## 6. Study Variables

### 6.1. Dependent/Outcome Variable

Knowledge, attitude, and health-seeking action of obstetric danger signs.

### 6.2. Independent Variables

Sociodemographic characteristics: maternal age, marital status, level of education, and occupation.

Obstetric characteristics: parity, gravidity, ANC attendance, and number of ANC visits.

### 6.3. Operational Definition

#### 6.3.1. Health-Seeking Actions

An individual undertakes sequence of remedial actions to rectify perceived ill-health. In this study, it refers to the health action a woman took after recognizing a danger sign during pregnancy, labor and delivery, and postpartum. The actions include doing nothing; consulting a friend/relative; self-care/treatment; consulting a TBA/traditional healer; and consulting a qualified health professional in a health facility. Healthcare-seeking actions were categorized as appropriate which is visiting a health facility or inappropriate which includes taking no action; consulting a friend/relative, or self-medication).

#### 6.3.2. Good Knowledge

A woman was considered knowledgeable if she spontaneously mentioned at least five key danger signs in any of the three phases of childbirth (pregnancy, childbirth, and postpartum) with at least one in each phase.

### 6.4. Data Processing and Analysis

The data gathered through the structured questionnaire was compiled, cleaned, coded, and entered into EpiData version 3.1 and exported to SPSS version 20 for analysis. Frequency was used to clean data. All the variables were entered into bivariate analysis; those explanatory variables with a *p* value <0.2 in crude analysis were considered as a candidate variable for multivariable analysis, and those variables with a *p* value <0.05 in multivariable analysis were considered as significant association. Backward stepwise was used. Strength of association was presented using odds ratios with 95% confidence intervals.

### 6.5. Ethical Considerations

Ethical clearance was obtained from the Wollega University Institute of Health Science Institutional Research Ethics Review Committee (IRERC), and informed consent from every participant was obtained before the interview. A formal letter of cooperation was written to the Nekemte Zone Health Office and respective kebeles. Participants were informed about the objective of the study and their rights that it will contribute necessary information for policymakers and other concerned bodies. Any involvement in the study was after their complete consent was obtained. Any woman who is not willing to participate in the study was not forced to participate. They were also informed that all data obtained from them would be kept confidential by using codes instead of any personal identifiers and are meant only for the purpose of the study. The interview was conducted in separate private places.

## 7. Results

### 7.1. Sociodemographic Characteristics of Participants

A total of 610 mothers were included in the study, yielding a response rate of 98.22% (610/621). The mean age of the study participants was 27.94 (SD ± 4.55). The majority, 83.4% (509/610), of respondents were ethnically Oromo, and the dominant religion was Protestant, 62.1% (379/610) (Table 1).

Among the respondents, 573 (93.9) of the study participants had at least one antenatal care (ANC) follow-up for their last pregnancy. The majority of the respondents, 284 (46.6%), start their ANC visit at 17–24 weeks of gestational age. Many mothers, 546 (89.5%), gave birth to their last child in the health facility (Table 2).

### 7.2. Obstetric Danger Sign Knowledge

From 610 respondents, only 32.3% (197/610) of respondents were able to spontaneously mention at least five obstetric danger signs in the three phases with at least one in each phase and thus were considered as having good knowledge of obstetric danger signs during pregnancy, intrapartum, and postpartum (Table 3).

### 7.3. Factors Associated with Obstetric Danger Sign Knowledge

In the bivariate analysis, age, gravidity, parity, occupation, educational status, ANC follow-up, and number of ANC visit were found to be significantly associated with obstetric danger sign knowledge. Multiple logistic regressions revealed that occupation, educational status, ANC follow-up, and number of ANC visits were variables significantly associated with knowledge of obstetric danger sign (Table 4).

This study revealed that government employees were 3.28 times more likely to have good knowledge of obstetric danger signs than housewives (adjusted OR = 3.28, 95% CI: 1.98–5.42). Mothers who were able to read and write were 4.92 times more knowledgeable about obstetric danger signs than those who were not able to read and write (adjusted OR = 4.92, 95% CI: 2.14–11.3). Mothers who have completed primary school were 4.90 times more knowledgeable about obstetric danger signs than those who were not able to read and write (adjusted OR = 4.90, 95% CI: 2.11–11.4). Mothers who had ANC follow-up were 6.2 times more knowledgeable about obstetric danger signs than those who have no ANC follow-up (adjusted OR = 6.2, 95% CI: 1.82–21.21). Mothers who had more than two ANC follow-ups were 4.07 more knowledgeable about obstetric danger signs than mothers having only less than two ANC visits (adjusted OR = 4.07, 95% CI: 2.35–7.06) (Table 4).

### 7.4. Healthcare-Seeking Actions after Recognition of Danger Signs

The healthcare-seeking actions were classified into two types: appropriate and inappropriate. A total of 24.6% (150/610) participants had experienced obstetric danger signs during their last pregnancy. From those who faced obstetric danger signs, the majority, 24.6% (150/610), of women, 91.3% (137/150), went to a health facility for care after they faced the obstetric danger signs. In this study, in those who had good knowledge about obstetric danger signs, the majority, 98.2%, of them had good practice seeking health facilities. The major reason mentioned by the respondents for not seeking health facility was poor knowledge of obstetric danger signs 92.3% (12/13) followed by distance away from health facility, 23.1% (3/13), and lack of transportation and lack of money 15.4% (2/13).

### 7.5. Attitude towards Obstetric Danger Signs

The majority, 96.6% (589/610), of the study respondents were agreed with the importance of knowing obstetric danger signs. The majority of the respondents, 96.2% (587/610), agreed that obstetric danger sign complication is preventable. And 16.9% (103/610) agreed that mothers who develop obstetric danger signs should seek help from traditional birth attendants.

## 8. Discussion

Obstetric danger sign knowledge is the first essential step in order to access and get appropriate and timely obstetric care. This study tried to assess factors affecting obstetric danger sign knowledge and health-seeking actions of women after identifying danger sign. The finding of this study indicated that 197 (32.3%) of the study participants were knowledgeable about key danger signs in the three phases with at least one in each phase that is during pregnancy, childbirth/labor, and postpartum. This finding is higher than the study done in Tanzania which was 25.2% [6]. This difference could be due to sociocultural differences in these study settings and the difference in the implementation of relevant health intervention programs.

Mothers' occupation showed significant statistical association with knowledge of obstetric danger signs. Government employees were 3.28 times more likely to have good knowledge of obstetric danger signs than housewives (adjusted OR = 3.28, 95% CI: 1.98–5.42). This is consistent with the study conducted in Goba District, Ethiopia [13] and Tanzania [6]. This might be because women who have their own income might have decision-making power to seek health care as compared to those who do not have their own income, and the majority of government employees had formal education. Educated women might have better access to health service information and can utilize information optimally.

Similarly, educational status showed statistical association with the mother's knowledge of obstetric danger signs. Mothers who were able to read and write were 4.92 times more knowledgeable about obstetric danger signs than those who were not able to read and write (adjusted OR = 4.92, 95% CI: 2.14–11.3). Mothers who were primary school were 4.90 times more knowledgeable about obstetric danger signs than who were not able to read and write (adjusted OR = 4.90, 95% CI: 2.11–11.4). This is similar to the previous study done in Tsegedie District, Tigray Region, Ethiopia [14], in Raya Kobo District of Ethiopia [15], and in KwaZulu-Natal, South Africa [7]. This might be because educated women may be more autonomous in deciding to seek health care and more empowered in accessing the health service information needed to act on the ANC advice about obstetric danger signs and as well may have improved perceptions of the causes of disease and treatment and can utilize the information optimally which could be an indication for intervention to encourage access of education for all women.

Having antenatal care and number of ANC visits indicated significant association with obstetric danger signs. Mothers who had ANC follow-up were 6.2 times more knowledgeable about obstetric danger signs than those who have no ANC follow-up (adjusted OR = 6.2, 95% CI: 1.82–21.21). Mothers who had more than two ANC follow-ups were 4.07 times more knowledgeable about obstetric danger signs than mothers having only less than two ANC visits. This is consistent with the study conducted in Raya Kobo District of Ethiopia [15] and contrast with the study done in Tanzania [6] where a number of ANC visits have no significant association. The difference could be due to quality of antenatal care and advice given at each visit which implies that appropriate advice and counseling during antenatal care is significant in improving knowledge of obstetric danger signs which in turn avoids delay to seek care, one of the major causes of maternal mortality.

In this study, 150 (24.6%) women had faced obstetric danger signs during their last pregnancy. From those who faced obstetric danger signs, the majority, 150 (24.6%), of them 137 (91.3%) had good practice seeking medical care when they faced obstetric danger signs. This is in line with the previous study done in Debre Berhan, Ethiopia, from 51 (8.1%) of the respondents who experienced obstetric danger signs, and 44 of them had good practice seeking medical care when they faced problems [16]. In this study, almost all mothers who had good knowledge about obstetric danger signs had good practice seeking health facilities after they faced obstetric danger signs. This indicates that if pregnant women recognize danger signs, they seek care and it can reduce the first delay to seek health care which in turn reduces maternal mortality.

The majority, 96.6% (589/610), of the study respondents agreed with the importance of knowing obstetric danger signs. This is comparable with the study conducted in Debre Berhan, Ethiopia [16]. The majority of the respondents, 96.2% (587/610), agreed that obstetric danger sign complication is preventable. And 16.9% (103/610) agreed that mothers who develop obstetric danger signs should seek help from traditional birth attendants. This is congruent with the study done in Debre Berhan, Ethiopia [16].

## 9. Conclusion

The knowledge and attitude of women regarding obstetric danger signs are not satisfactory to have healthy pregnancy and child birth. However, the practice of mothers in response to obstetric danger signs was boosting despite poor knowledge and attitude. Educational status, occupation, having ANC follow-up, and number of ANC visits were found to be factors significantly associated with obstetric danger sign knowledge.

### 9.1. Limitations

As a cross-sectional study requires respondents to remember information retrospectively, and recall bias and lack of the literature for attitude and health-seeking actions were the potential limitation of this study.

### 9.2. Recommendations

Generally, this study showed that a significant proportion of women did not have awareness about obstetric danger signs which implies that the majority of mothers who lack awareness are likely to delay in deciding to seek health care. Therefore, concerned bodies should design appropriate strategies including the provision of targeted health education or provide information, education, and communication to pregnant mothers to increase their awareness and thus enable early recognition of serious health problems during pregnancy, labor, and postpartum.

## Figures and Tables

**Table 1:** Sociodemographic characteristics of respondents in Nekemte Town, West Ethiopia, November 2017.

Variable	Frequency (*N* = 610)	Percentage (%)
Age		
≤20	23	3.8
21–25	170	27.9
26–30	255	41.8
>30	162	28.6

Ethnicity		
Oromo	509	83.4
Amhara	65	10.7
Gurage	31	5.1
Others	5	0.8

Religion		
Protestant	379	62.1
Orthodox	176	28.9
Muslim	45	7.4
Wakeffata	10	1.6

Marital status		
Married	577	94.6
Divorced	18	3
Widowed	9	1.5
Unmarried	6	1

Occupation		
Housewife	243	39.8
Government employee	143	23.4
Merchant	138	22.6
Daily laborer	80	13.1
Others	6	1

Educational status		
Unable to read and write	60	9.8
Able to read and write	127	20.8
Primary	113	18.5
Secondary	125	20.5
College and above	185	30.3

Household income		
≤500	29	4.8
501–1000	106	17.4
1001–1500	78	12.8
>1500	397	65.1

**Table 2:** Obstetrical characteristics of respondents in Nekemte Town, West Ethiopia, November 2017.

Variables	Frequency (*N* = 369)	Percentage (%)
Gravidity		
1	119	19.5
2–4	483	79.2
≥5	8	1.3

Parity		
1	125	20.5
2–4	479	78.5
≥5	6	1

Antenatal care visit (ANC)		
Yes	573	93.9
No	37	6.1

Frequency of ANC visit		
1	47	8.2
2	90	15.7
3	259	45.2
≥4	177	30.9

Gestational age during first ANC visit		
≤16	220	36.1
17–24	284	46.6
25–32	66	10.8
≥33	3	0.5

Place of delivery		
Home	64	10.5
Health facility	546	89.5

**Table 3:** Knowledge of obstetric danger signs among postnatal mothers in Nekemte Town, West Ethiopia, November 2017 (*N* = 610).

Variable	Frequency (^*∗∗*^)	Percentage (%)
Key danger signs during pregnancy		
(i) Vaginal bleeding	243	39.8
(ii) Sudden gush of fluid before labor	101	16.6
(iii) Severe headache	205	33.6
(iv) Blurred vision	196	32.1
(v) Excessive vomiting	61	10
(vi) Swelling of hands/face	106	17.4
(vii) Loss of fetal movement	110	18
(viii) Premature onset of contraction	68	11.1
(ix) Severe unusual abdominal pain	58	9.5

Key danger signs during delivery		
(i) Severe vaginal bleeding	15	2.5
(ii) Prolonged labor	56	9.2
(iii) Convulsions	5	0.8
(iv) Retained placenta	19	3.1

Key danger signs during postpartum		
(i) Severe bleeding following childbirth	18	3
(ii) Loss of consciousness after childbirth	5	0.8
(iii) Fever	39	6.4
(iv) Foul-smelling vaginal discharge	25	4.1

Did not mention any danger signs	157	25.7
Mentioned at least 5 danger signs with at least one in each danger sign	197	32.3

^*∗∗*^Multiple responses were possible.

**Table 4:** Bivariate and multivariate analysis of factors associated with knowledge of obstetric danger signs among postnatal mothers in Nekemte Town, November 2017.

Variable	Poor knowledge *N* [%]	Good knowledge *N* [%]	COR [95% CI]	AOR [95% CI]
Age				
≤20	10 [43.5]	13 [56.5]	1	**1**
21–25	100 [58.8]	70 [41.2]	4.1 [1.67–10.1]^*∗*^	0.41 [0.15–1.1]
26–30	180 [70.6]	75 [29.4]	2.2 [1.38–3.54]^*∗*^	0.25 [0.09–1.2]
>30	123 [75.9]	39 [24.1]	11.3 [0.84–2.1]	0.18 [0.07–1.3]

Gravidity				
1	64 [53.8]	55 [46.2]	1	1
2–4	343 [71]	140 [29]	10.48 [0.32–0.72]^*∗*^	1.1 [0.29–4.2]
≥5	6 [75]	2 [25]	0.39 [0.08–2.0]	4.2 [0.19–9.5]

Parity				
1	67 [53.6]	58 [46.4]	1	1
2–4	341 [71.2]	138 [28.8]	0.47 [0.32–0.7]^*∗*^	0.65 [0.17–2.4]
≥5	5 [83.3]	1 [16.7]	0.23 [0.03–2.04]^*∗*^	0.12 [0.003–5.01]

Occupation				
Housewife	161 [72.9]	60 [27.1]	1	
Government employee	106 [54.4]	89 [45.6]	2.25 [1.41–3.39]^*∗*^	3.28 [1.98–5.42]^*∗*^
Merchant	88 [69.3]	39 [30.7]	1.19 [0.74–1.92]	1.27 [0.76–2.14]
Daily laborer	58 [86.6]	9 [13.4]	0.42 [0.19–0.89]	0.47 [0.21–1.04]

Educational status				
Unable to read and write	56 [86.2]	9 [13.8]	1	1
Able to read and write	73 [57.5]	54 [42.5]	4.6 [2.09–10.1]^*∗*^	4.92 [2.14–11.3]^*∗*^
Primary	62 [55.4]	50 [44.6]	5.02 [2.26–11.13]^*∗*^	4.90 [2.11–11.41]^*∗*^
Secondary	91 [73.4]	33 [26.6]	2.26 [1.01–5.07]^*∗*^	2.15 [0.89–5.14]
College and above	131 [72]	51 [28]	2.42 [1.12–5.26]^*∗*^	1.31 [0.51–3.26]

ANC follow-up during pregnancy				
No	34 [91.9]	3 [8.1]	1	1
Yes	379 [66.1]	194 [33.9]	5.8 [1.76–19.13]^*∗*^	6.2 [1.82–21.2]^*∗*^

Number of ANC visit				
≤2 times	118 [86.1]	19 [13.9]	1	1
>2 times	261 [59.9]	175 [40.1]	4.2 [2.47–7.0]^*∗*^	4.07 [2.35–7.06]^*∗*^

^**1**^Reference. ^*∗*^Significant association.

## Data Availability

Data will be available on request from the corresponding author.

## Abbreviations

ANC: Antenatal care
IRERC: Institutional Research Ethics Review Committee
MOH: Ministry of Health
TBA: Traditional birth attendant.

## Supplementary Materials

Supplementary Materials This section includes questionnaires to assess obstetric danger signs.
