# Supplementary material for: Obstetric Danger Signs: Knowledge, Attitude, Health-Seeking Action, and Associated Factors among Postnatal Mothers in Nekemte Town, Oromia Region, Western Ethiopia—A Community-Based Cross-Sectional Study
Source: Obstet Gynecol Int. 2020 Sep 1;2020:6573153. doi: 10.1155/2020/6573153 (PMC7481917; doi:10.1155/2020/6573153)
Supplement: Supplementary Materials — This section includes questionnaires to assess obstetric danger signs. [file 6573153.f1.docx]

Questionnaires to **assess Obstetric Danger Signs: Knowledge, Attitude, Health seeking behavior and Associated factors among Postnatal Mothers in Nekemte Town and surrounding Kebele, Oromia Region, Western Ethiopia 2017: Community Based Cross-Sectional Study**

If Respondent agrees to be interviewed, starting time___________: End time_________:

001. Questionnaire Code __________

002. How long you have been living here?

1. 12 months and above 2. Less than 12 months -----go to next house

Date of data collection________/_________/_________

Name of data collector__________________________ signature_______________

Name of supervisor_____________________________ signature_______________

**Part I: SOCIODEMOGRAPHIC AND OBSTETRIC CHARACTERISTICS**

| **Sr.no** | **Questions** | **Choice answers** | **Skip to ___** |
| --- | --- | --- | --- |
| 101 | Age | in years**_______** |  |
| 102 | Marital Status: | 1. Married  2. Divorced  3. Widowed  4. single |  |
| 103 | Religion | 1.Protestant  2.Orthodox  3.Muslim  4.Waaqeffataa  5.Others specify_____________ |  |
| 104 | Ethnicity | 1. Oromo  2. Amhara  3.Gurage  4. Others, specify_____ |  |
| 105 | occupation | 1. House wife  2. Gov’t Employee  3. farmer  4. merchant  5. daily labourer  6.Others Specify______ |  |
| 106 | Educational status of the mother | 1. unable to read and write 2. Able to read and write 3. Primary school 4. Secondary school 5. College and above |  |
| 107 | Average monthly family income | in birr_________ |  |

**Part 2:** **Obstetric** **characteristics**

| **Sr.no** | **Questions** | **Choice answers** | **if no Skip to** |
| --- | --- | --- | --- |
| 201 | Gravida | **___________** |  |
| 202 | Para | **___________** |  |
| 203 | Have you attended ANC follow up when you were pregnant? | 1.Yes  2.No | **If no skip to 208** |
| 204 | Place of Antenatal visits | 1. Governmental hospital 2. Private hospital / Clinic 3. Health center |  |
| 205 | If yes Number of Visits | - 1. Visit   2.2 visit  3.3 visit  4.>=4 visit |  |
| 206 | What was the age of pregnancy at your first ANC visit? | _____________ In weeks |  |
| 207 | In any of those ANC visits did you get an opportunity to be advised/counselled on the  Following; more than one redponse is possible | 1. Where to deliver 2. Benefits of delivering at the health facility/hospital 3. What to do in case of any complication |  |
| 208 | Where did you gave birth your last child/birth? | 1.Home  2.Health facility |  |
| **Part III: KNOWLEDGE ON Obstetric DANGER SIGNS** | | | |
| 209 | Have you ever heard or have Information about Obstetric danger signs? | 1. Yes 2. No |  |
| 2010 | If Yes, which danger sign do you know ? | ***Danger signs During pregnancy**   1. Vaginal bleeding 2. Sudden gush of fluid before labor 3. Severe head ache 4. Dizziness and blurred vision 5. Excessive vomiting 6. Swelling of hands, face 7. Loss of fetal movement 8. Premature onset of contraction 9. Severe unusual abdominal pain   * **danger signs during labor and childbirth.**  1. severe vaginal bleeding,  2.prolonged labor  3.convulsions  4. retained placenta.  **Danger signs during the postpartum period**   1. severe bleeding following childbirth 2. loss of con-sciousness after childbirth 3. fever. 4. Foul smelling vaginal discharge |  |
| 211 | Where did you hear about danger signs during pregnancy? | 1. Family member  2. Neighbor  3. health institution  4.mass media |  |
| **Part IV: Health seeking behavior** | | | |
| 2015 | Have you ever faced any Obstetric danger signs in your last pregnancy? | 1.Yes  2.No | **If no skip to 2017** |
| 2016 | If yes to question number 2015 which one you faced? | ***Danger signs During pregnancy**  1.Vaginal bleeding  2.Sudden gush of fluid before labor   1. Severe head ache 2. Dizziness and blurred vision 3. Excessive vomiting 4. Swelling of hands, face 5. Loss of fetal movement 6. Premature onset of contraction 7. Severe unusual abdominal pain (Epigastric pain)   * **danger signs during labor and childbirth.**  1. severe vaginal bleeding,  2.prolonged labor,  3.convulsions,  4. retained placenta.  **Danger signs during the postpartum period**   1. severe bleeding following childbirth 2. loss of con-sciousness after childbirth 3. fever. 4. Foul smelling vaginal discharge |  |
| 215 | What was your actions when you faced the Obstetric danger signs in your last pregnancy? | 1.Nothing  2.Consulted a friend/relative  3Self-care/treatment  4Consulted a  TBA/traditional healer  5. Went to a health facility  6.Others(specify)______________ | **If 1,2,3,4 & 6 skip to 2017** |
| 2016 | If you went to health facility  how many hours /days you take to seek health care provider/Health facility? | 1. 1-4Hrs 2. 5-8Hrs 3. One day 4. Two days   More than two days |  |
| 2017 | Reason for delayed in health care  seeking | 1. Lack of awareness about obstetric danger sign 2. Health Center is far 3. Lack of money 4. Lack of transportation |  |
| **Part V: Attitude towards obstetric danger signs** | | | |
| 2018 | Do you agree importance of knowing obstetric danger signs | 1.yes  2.No |  |
| 2019 | Do you agree obstetric danger sign is preventable? | 1.yes  2.No |  |
| 220 | Do you agree on the idea that mothers who develop obstetric danger signs should seek help from traditional birth attendants. | 1.yes  2.No |  |
| 221 | Do you agree on the idea that mothers who develop obstetric danger signs should seek help from other older women. | 1.yes  2.No |  |

**Afan Oromoo Version**

Questionnaires to **assess Obstetric Danger Signs: Knowledge, Attitude, Health seeking behavior and Associated factors among Postnatal Mothers in Nekemte Town and surrounding Kebele, Oromia Region, Western Ethiopia 2017: Community Based Cross-Sectional Study**

**UNKA WAADA**

Maqaan kiyya ____________________jedhama.kanin dhuufee garee Barsiisota wallaggaa Yuunivarsitiin qorannoo geggeeffamaa jiruuf odeeffannoo funaanuuf yoo ta’u, kayyoon qorannoo Kanaas hubannoo Haadholiin waggaa tokko darbe kana keessatti dahan mallattoolee balaafamoo yeroo ulfaa,da’umsaafi da’umsa booda miidhaa geessisan irratti qaban,Ilaalchaafi sababoota hubanno kanaa wajjin wal qabataniif amala yaalii barbaaduu irratti qaban qoracuufi. Odeeffannon isin kennitan hundi iccitiidhaan qabama. Maqaa fi iddoo jireenya keessanii barreessuun hin barbaachisu. Qorannoo kana keesatti hirmaachuun fedhi irratti kan hundaa’ee ta’a. Hirmaachuu fi dhisuuf mirgi keessan kan kabajamee dha. Haata’u malee galmaan ga’umsa kaayyoo qoranichaatiifii fooya’insa tajaajila fayyaa haadholiif jecha hirmaanaan keessan murteesaa waan ta’eef akka gaafileedhaaf deebi kennudhaan hirmaattan kabajaan isin gaafadha. Kanaaf qorannoo kana keessatti hirmaachuuf fedhii keessanii?

1.Eyyee 2.Lakki-----------yoo ta’e gara mana itti aanuutti darbi

Yoo walii galan , Sa’a itti eegale___________: sa’a itti xumurame_________:

002. Hammamiif as jiraatte?

1. Ji’a jahaafi isaa ol 2. Ji’a jahaa gad -----gara mana itti aanuutti darbi.

Guyyaa odeeffannoon funaaname________/_________/_________

Maqaa nama odeeffannoo funaanee __________________________ Mallattoo______________

Maqaa to’ataa _____________________________ Mallattoo_______________

**Qajeelcha:Gaaffiwwan armaan gadiif itti maruun deebisi**

**Kutaa tokkoffaa: Gaaffiwwan Aadaafi Hawaasummaa ilaalan**

| **Tartiiba**  **Lakk.** | **Gaaffii** | **Filanno deebii** | **Gara __darbi** |
| --- | --- | --- | --- |
| 101 | Umuriinkee meeqa? | Waggaadhaan.**_______** |  |
| 102 | Haalli Gaa’elakee akkam amma? | 1. Kan heerumte  2. Kan hiikte  3. Kan irra du’e  4. Kan hin heerumne |  |
| 103 | Amantiinkee maali? | 1.Pirotestaantii  2.Ortodoksii  3.Islaamaa  4.Waaqeffataa  5.Kan biraa ibisi____________ |  |
| 104 | Qomoonkee maali? | 1. Oromoo  2. Amaaraa  3.Guraagee  4. Kan biraa ibsi_____ |  |
| 105 | Hojiinkee maali? | 1. Giiftii Manaa  2. Hojjetttuu Mootummaa  4. Daldaltuu  5. Hojjettuu Guyyaa  6.Kan biraa ibsi______ |  |
| 106 | Sadarkaa Barumsaakee hagam? | 1. Barreessuuf dubbisuu kan hin dandeenye 2. Barreesuuf Dubbisuu Kan dandeessuu 3. Sadarkaa tokkoffaa 4. Sadarkaa lammaffaa 5. Kolleejjiifi isaa ol |  |
| 107 | Galiin Maatiikee Ji’aan hagam? | Qarshii_________ |  |
| 108 | Qabeenya Maatiikee ollaakee wajjin yoo madaaltee maal fakkaataa? | 1.Baayyee sooressa  2. Sooressa  3. Hiyyeessa  4.Baayyee Hiyyeessa |  |

**Kutaa lammaffaa:** **Gaaffiilee Da’umsa ilaalan**

| **Tartiiba**  **Lakk.** | **Gaaffii** | **Filannoo Deebii** | | **Lakki yoo ta’e gara__darbi** | | |
| --- | --- | --- | --- | --- | --- | --- |
| 201 | Yeroo Meeqa ulfoofte/Gravidity? | **___________** | |  | | |
| 202 | Yeroo Meeqa Deesse/Parity? | **___________** | |  | | |
| 203 | Yeroo ulfa turte hordoffii ulfaa gooteettaa? | 1.Eeyyee  2.Lakkii | | **If no skip to 208** | | |
| 204 | 203 Eeyyee yoo ta’e eessaatti hordofamaa turte? | 1. Hospitaala Mootummaa 2. Kilinika dhuunfaa 3. Buufata fayyaa | |  | | |
| 205 | Yeroo meeqa ilaalamte? | 1. yeroo tokko 2. Yeroo lama 3. Yeroo sadii 4. Yeroo afuriif isaa ol | |  | | |
| 206 | Yeroo jalqaba ilaalamuuf gara mana yaalaa deemte ulfikee torban meeqa turee? | _____________ torbaniin | |  | | |
| 207 | Yeroo hordoffii ulfaa godhaa turtetti gorsi siif kenname maali/jiraa? tokkoo ol deebisuun ni danda’ama | 1. Eessatti da’uu akka qabdu 2. Bu’aa mana yaalaatti da’uun qabu 3. Rakkoon yoo si mudate maal gochuu akka qabdu | |  | | |
| 208 | Mucaakee yeroo darbee/isa dhumaa eessatti deesse? | 1.Mana  2.Mana yaalaa | |  | | |
| **Kutaa sadaffaa:Hubannoo Haadholiin qaban** | | | | | | |
| 209 | Waa’ee mallattoolee balaafamoo yeroo ulfaa,da’umsaa fi da’umsa booda miidhaa geessisanii dhageesse beektaa? | 1. Eeyyee 2. Lakkii | |  | | |
| 2010 | Eeyyee yoo ta’e mallattoo balaafamoo kamiin beekta ?tokkoo ol deebisuun ni danda’ama | ***Mallattoolee yeroo ulfaa miidhaa geessisan**   1. kara gadameessaa Dhiiguu 2. Bishaan gubbee ciniinsuu dura dhangala’u 3. Mata dhukkubbii gar-malee 4. Ija dura maruu 5. Balaqqamsiisa garmalee 6. Dhidhiita’uu fuulaaf harkaa 7. Sochii mucaa dhabuu 8. Utuu ji’i hin ga’in ciniinsuun jalqabuu 9. Laphee/qoma jala garmalee dhukkubuu   ***Mallattoolee yeroo Da’umsaa miidhaa geessisan**  1. Kara gadameessaa garmalee dhiiguu  2. Ciniinsuun irra turuu  3.hurgufamuu qaamaa/**convulsions**  4, Oofkaltiin bahuu diduu.  ***Mallattoolee da’umsa booda miidhaa geessisan**  1.Kara gadameessaa garmalee dhiiguu  2.Of wallaaluu/loss of consciousness  3.Dhaqna gubaa /Fever  4.Dhangal’aan qaama keessaa ba’uu foolii godhachuu | |  | | |
| 211 | Mallattoolee balaafamoo yeroo ulfaa,da’umsaa fi da’umsa booda miidhaa geessisan kana eessaa dhageesse/**source of information**? | 1. Miseensa maatiirraa  2. Ollaarraa  3. Mana yaalaa  4.Meshaalee sub-quunnamtii/**mass media** | |  | | |
| **Kutaa afuraffaa: Amala /shakala haadholiin mana yaalaa deemuu irratti qaban** | | | | | | |
| 2015 | | Mallattoolee balaafamoo yeroo ulfaa da’umsaa fi da’umsa booda miidhaa geessisuu danda’an kunnin si mudatee beekaa? | | 1.Eeyyee  2.Lakki | | Lakkii yoo ta’e gara 2017 darbi |
| 2016 | | Yoo si mudatee beeka ta’e isa kamtu si mudatee? | | ***Mallattoolee balaafamoo yeroo ulfaa miidhaa geessisan**   1. kara gadameessaa Dhiiguu 2. Bishaan gubbee ciniinsuu dura dhangala’u 3. Mata dhukkubbii gar-malee 4. Ija dura maruu 5. Balaqqamsiisa garmalee 6. Dhidhiita’uu fuulaaf harkaa 7. Sochii mucaa dhabuu 8. Utuu ji’i hin ga’in ciniinsuun jalqabuu 9. Laphee/qoma jala garmalee dhukkubuu   ***Mallattoolee balaafamoo yeroo Da’umsaa miidhaa geessisan**  1. Kara gadameessaa garmalee dhiiguu  2. Ciniinsuun irra turuu  3.hurgufamuu qaamaa/**convulsions**  4, Oofkaltiin bahuu diduu.  ***Mallattoolee balaafamoo da’umsa booda miidhaa geessisan**  1.Kara gadameessaa garmalee dhiiguu  2.Of wallaaluu/loss of consciousness  3.Dhaqna gubaa /Fever  4.Dhangal’aan qaama keessaa ba’uu foolii godhachuu | |  |
| 215 | | Yeroo mallattooleen balaafamoon miidhaa geessisuu danda’an kunniin si mudatan maal goote ture? | | 1.Homaa  2.Fira/hiriyaattin hime  3ofiikoon of yaale  4deessistoota aadaa biran deemee  5. Gara mana yaalaan deemee  6.Kan biraa ibsi______________ | | **yoo 1,2,3,4 & 6 gara 2017** |
| 2016 | | Yoo gara mana yaalaa deemte ta’e yeroo hagamii keessatti deemtee? | | 1. Sa’a 1-4 keessatti 2. Sa’a 5-8 Keessatti 3. Guyyaa tokko keessatti 4. Guyyaa lama fi isaa ol booda | |  |
| 2017 | | Yoo mana yaala hin deemne ta’e maalif? | | 1. Hubannoo waan hin qabneef 2. Mana yaalaa fagoo waan ta’eef 3. Qarshii waan hin qabneef 4. Tiraanispoortii dhabeen | |  |
| **Kutaa shanaffaa:Ilaalcha haadholeen qaban** | | | | | | |
| 2018 | | Mallattoolee balaafamoo yeroo ulfaa,da’umsaafi da’umsa booda miidha geessisan beekuun barbaachisaadha jettee yaaddaa? | | 1.Eeyyee  2.Lakkii | |  |
| 2019 | | Mallattoolee balaafamoo yeroo ulfaa,da’umsaa fi da’umsa booda miidhaa geessisan ittisuun ni danda’ama jettee yaaddaa? | | 1.Eeyyee  2.Lakkii | |  |
| 220 | | Haadholeen mallattoolee balaafamoo kun mudatan gara deessistoota aadaa deemuu qabu jettee ni amantaa? | | 1.Eeyyee  2.Lakkii | |  |
